# Factors associated with gastrointestinal side effects after liraglutide treatment for type 2 diabetes

**DOI:** 10.3389/fendo.2023.1098032

**Published:** 2023-01-30

**Authors:** Hao Wu, Zongshi Lu, Runyao Chen, Quanfang Cai, Miaomiao Wang, Liting Zhang, Zhiming Zhu

**Affiliations:** ^1^ Department of Endocrinology, the 910th Hospital of Chinese People's Liberation Army, Quanzhou, China; ^2^ Department of Hypertension and Endocrinology, Daping Hospital, Army Military Medical University, Chongqing, China

**Keywords:** liraglutide, gastrointestinal side effects, type 2 diabetes mellitus, risk factors, thyroid stimulating hormone

## Abstract

**Aim:**

To identify risk factors predictive of or associated with gastrointestinal side effects (GISE) of liraglutide in patients with type 2 diabetes (T2DM).

**Methods:**

T2DM patients treated with liraglutide for the first time were obtained and grouped into patients without GSEA and patients with GSEA. Baseline variables, including age, sex, body mass index (BMI), glycemia profiles, alanine aminotransferase, serum creatinine, thyroid hormones, oral hypoglycemic drugs and history of gastrointestinal diseases, were tested for possible associations with GSEA outcome. Significant variables were entered into univariate and multivariate logistic regression (forward LR) analyses. Receiver operating characteristic (ROC) curves to determine clinically useful cutoff values.

**Results:**

A total of 254 patients (95 female) were included in this study. 74 cases (29.13%) reported GSEA and 11 cases (4.33%) discontinued treatment. The results of univariate analyses showed that sex, age, thyroid stimulating hormone (TSH), free triiodothyronine, α-glucosidase inhibitor (AGI), and concomitant gastrointestinal diseases were associated with GSEA occurrence (all p <0.05). In the final regression model, AGI use (adjusted OR=4.01, 95%CI: 1.90-8.45, p<0.001), gastrointestinal diseases (adjusted OR=3.29, 95%CI: 1.51-7.18, p=0.003), TSH (adjusted OR=1.79, 95%CI: 1.28-2.50, p=0.001) and male sex (adjusted OR=0.19, 95%CI: 0.10-0.37, p<0.001) were independently associated with GSEA. Furthermore, ROC curve analysis confirmed that TSH values of 1.33 and 2.30 in females and males, respectively, were useful thresholds for predicting GSEA.

**Conclusion:**

This study suggests that the combination of AGI, concomitant gastrointestinal diseases, female sex and higher TSH levels are independent risk factors of GSEA of liraglutide treatment in patients with T2DM. Further research is warranted to elucidate these interactions.

## 1 Introduction

The prevalence of diabetes in China increased from 10.9% in 2013 to 12.4% in 2018, with approximately 140 million people suffering from diabetes (1). Liraglutide, a novel glucose-lowering drug, has been extensively used in clinical practice for the treatment of type 2 diabetes mellitus (T2DM) since approved by the US Food and Drug Administration (FDA) in 2010 (2). The pharmacological property of liraglutide is to mimic the actions of the incretin hormone glucagon-like peptide 1 (GLP-1), which produces a variety of hypoglycemic effects on multiple target organs, including an increase in glucose-stimulated insulin secretion, suppression of hepatic glucagon secretion, deceleration of gastric emptying, promotion of satiety and reduction of calorie intake (3, 4). Although seven GLP-1 receptor agonists are available in China, liraglutide is a preferred choice for patients with T2DM partly because it can be partially covered by health insurance.

Liraglutide injection once daily at a dose of 1.2 to 3.0 mg not only lowers glycated hemoglobin (HbA1c) and fasting blood glucose but also has benefits in reducing weight and improving the cardiovascular and renal function of T2DM (5–7). Particularly, liraglutide at a 3.0 mg daily dose was approved by both the FDA and European Medicine Agency as adjunct to a comprehensive lifestyle intervention for the management of overweight/obesity. Liraglutide may be a potential drug for the treatment of Alzheimer’s disease by reducing neuroinflammation and oxidative stress (8). We have also recently observed improved cognitive decline in T2DM patients treated with liraglutide, which was independent of its hypoglycemic effect and weight loss (9). However, liraglutide often causes gastrointestinal side effects (GSEA) in patients during the titration period, with typical symptoms including nausea, vomiting, diarrhea and abdominal pain (10). GSEA is also the most common cause of withdrawal from liraglutide treatment.

Several large randomized controlled trials reported that the incidence of GSEA of liraglutide in patients with T2DM was 20% to 40% (11–13). Notably, the gastrointestinal adverse events of liraglutide were as high as 60% to 80% in overweight adolescents without diabetes (14, 15). Although most patients tolerate mild or moderate GSEA because these side effects are typically transient and usually subside after a few weeks of treatment, it still causes 4% to 5% of patients to discontinue liraglutide treatment due to severe or persistent gastrointestinal adverse reactions according to Liraglutide Effect and Action in Diabetes (LEAD) trials (11). A higher rate of discontinuation was observed in real-world settings, and 45.2% of patients with T2DM discontinued GLP-1 receptor agonists therapy at 12 months in the UK (16). Thus, exploring the factors affecting the GSEA of liraglutide and taking precautions accordingly may increase patient adherence to liraglutide.

A study found that the GSEA of liraglutide is related to age and metformin use (17), but there is a lack of other studies to validate these findings, and it remains unknown whether the other risk factors also influence the GSEA. In this retrospective study, we further investigated some identifiable risk factors for developing GSEA among patients treated with liraglutide.

## 2 Methods

### 2.1 Subjects

The data are from patients with type 2 diabetes who were hospitalized for the management of hyperglycemia at 910^th^ Hospital of PLA, China, from January 2021 to June 2022 and treated with liraglutide (18 mg/3 ml from Novo Nordisk, Denmark) for the first time. The initial dose of liraglutide was 0.6 mg/day, and the dose was adjusted to 1.2 mg/day after one week, or increased to reach a maximum dose of 1.8 mg/day in weekly intervals by 0.6 mg when hyperglycemia was uncontrolled. Previous treatments with the oral antidiabetic drug dose and/or combination with insulin were continued unchanged. No obvious gastrointestinal symptoms were observed in the patient prior to the initiation of liraglutide. The diagnosis of T2DM was based on the American Diabetes Association criteria with a glycated hemoglobin (HbA1c) value of >6.5% or fasting plasma glucose (FPG) >7.0 mmol/L (18). Contributors were asked to report on the occurrence of any GSEA or other possible treatment-related adverse events, as well as the main reason for liraglutide discontinuation if this occurred. The symptoms of GSEA were defined as those of nausea, vomiting, diarrhea, crampy abdominal pain, reflux, flatulence or similar related terms. The exclusion criteria included the following: (1) T2DM with acute diabetic complications; (2) Type 1 diabetes mellitus; (3) malignancy, e.g., thyroid cancer and gastric cancer; (5) thyroid dysfunction still taking thyroid hormone replacement therapy or drugs that inhibit thyroid hormone synthesis; (6) GSEA caused by drugs other than liraglutide; and (7) unwillingness or inability to provide information about GSEA.

### 2.2 Study outcomes and risk variables

The primary outcome was whether there were gastrointestinal side effects within 4 weeks after treatment with liraglutide. According to the GSEA outcome, patients were classified into two groups: a) patients without GSEA and b) patients reporting GSEA. The clinical parameters of patients, including age, sex, body mass index (BMI), glycemia profile (HbA1c, fasting insulin and C-peptide), alanine aminotransferase, serum creatinine and thyroid function, were collected before treatment with liraglutide. Oral hypoglycemic drugs (metformin and/or alpha-glucosidase inhibitors) and history of gastrointestinal diseases (esophagitis, gastritis, gastroesophageal reflux, enteritis, etc.) were also recorded.

### 2.3 Statistical analysis

All statistical analyses were performed using SPSS 26.0 (SPSS Inc., Chicago, USA) or R software 3.6.3. Continuous variables are described using the mean ± standard deviation (SD) for normally distributed variables and medians with interquartile range (IQR) for nonnormally distributed data. Continuous variables were tested between the GSEA group and the non-GSEA group using Student’s t test for normally distributed variables and the Mann−Whitney U test for nonnormally distributed variables. Categorical variables were tested using Fisher’s exact test. Variables with a statistically significant association in univariate analyses (p<0.05) were entered into a binary univariate logistic regression analysis, and variables with a p value (p<0.05) were subsequently entered into multivariate stepwise logistic regression (forward LR) to achieve a final model. Receiver operating characteristic (ROC) curve analysis was performed by the OptimalCutpoints package in R software to determine the area under the curve (AUC) and cutoff values. The mediation effects of TSH were analyzed by PROCESS SPSS version 4.0 software (19) with a simple mediation model (Model No.4). The Bootstrapping method with 5000 samples was used to generate the 95% confidence interval for the mediating effects. A two-tailed p value <0.05 was considered statistically significant.

## 3 Results

### 3.1 Clinical characteristics of the patients

A total of 296 T2DM patients treated with liraglutide were enrolled during the study period, and 254 cases were entered into the analysis according to the inclusion and exclusion criteria. Baseline characteristics are provided in **Table 1**. The patients included 159 males (62.60%) and 95 females (37.40%), with an age of 52.20 ± 12.61 years, body mass index (BMI) of 25.33 ± 3.97 kg/m^2^ and HbA1c of 10.06 ± 2.34%. A total of 221 (87.01%) and 56 (22.05%) patients were treated with metformin and an α-glucosidase inhibitor, respectively. Comorbidities of gastrointestinal diseases were observed in 44 (17.32%) patients. Seventy-four (29.13%) patients reported GSEA, of whom 11 (4.33%) discontinued liraglutide treatment due to intolerable GSEA within 4 weeks.

**Table 1:** Baseline characteristics and their association with reporting gastrointestinal side effects (GSEA) among patients treated with liraglutide.

Characteristic	All (n=254)	GSEA (n=74)	non-GSEA (n=180)	*p* value
Age(year)	52.20 ± 12.61	54.78 ± 11.42	51.14 ± 12.95	0.036*
Male (female)	159 (95)	28 (46)	131 (49)	< 0.001*
BMI (kg/m2)	25.33 ± 3.97	24.83 ± 4.11	25.53 ± 3.90	0.198
HbA1c (%)	10.06 ± 2.34	10.12 ± 2.61	10.03 ± 2.22	0.780
FISN (uIU/ml)	6.83 (3.71,13.00)	6.46 (3.58,12.24)	6.97 (3.71,13.25)	0.675
C-peptide (ng/mL)	1.94 (1.29,2.80)	1.82 (1.27,2.63)	1.97 (1.29,2.83)	0.643
TSH (uIU/ml)	1.58 (1.04,2.16)	1.85 (1.30,2.73)	1.5 (1.01,2.12)	0.004*
FT3 (pmol/L)	4.08 ± 0.62	3.94 ± 0.60	4.13 ± 0.63	0.029*
FT4 (pmol/L)	15.19 ± 3.21	14.88 ± 3.06	15.32 ± 3.27	0.321
ALT (U/L)	23.25 (16.08,39.50)	21.45 (14.85,34.95)	24.75 (16.65,41)	0.118
SCr (μmol/L)	57.30 (48.58,72.15)	55.00 (43.58,69.40)	59.05 (49.93,74.6)	0.065
metformin Yes (No)	221 (33)	65 (9)	156 (24)	1.000
AGI Yes (No)	56 (198)	25 (49)	31 (149)	0.007*
GID Yes (No)	44 (210)	19 (55)	25 (155)	0.004*

Variables are shown as the mean ± sd or median (interquartile range).

*BMI*, body mass index; *HbA1c*, glycosylated hemoglobin; *FISN*, fasting plasma insulin; *TSH*, thyroid stimulating hormone; *FT3*, free triiodothyronine; *FT4*, free thyroxine; *ALT*, alanine aminotransferase; *SCr*, serum creatinine; *GID*, gastrointestinal diseases; *AGI*, α-glucosidase inhibitor. *p < 0.05 (GSEA vs. non-GSEA).

### 3.2 Comparisons of clinical variables

The results of univariate analyses in **Table 1** showed that sex, age, TSH, FT3, α-glucosidase inhibitor (AGI), and concomitant gastrointestinal diseases were significantly associated with the occurrence of GSEA (all p <0.05). However, variables including BMI, HbA1c, fasting insulin, C-peptide, alanine aminotransferase, serum creatinine, FT4 and the use of metformin were not significantly associated with the GSEA of liraglutide (all p>0.05).

### 3.3 Risk factors predictive or associated with GSEA

**Figure 1:**
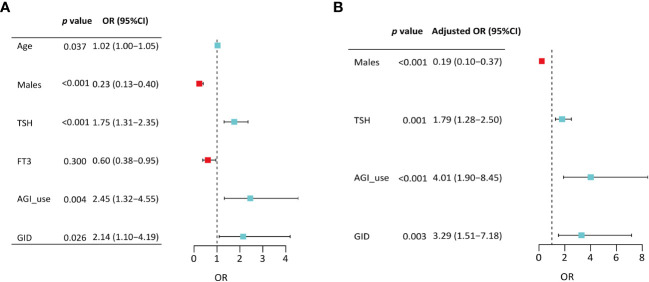
Binary logistic regression analyses of variables associated with gastrointestinal side effects among patients treated with liraglutide. **(A)** Model from all significant variables in univariate analyses; **(B)** Final model from after sequentially removing variables with the nonsignificant P value. TSH, thyroid stimulating hormone; FT3, free triiodothyronine; AGI, α-glucosidase inhibitor; GID, gastrointestinal disease.

**Figure 2:**
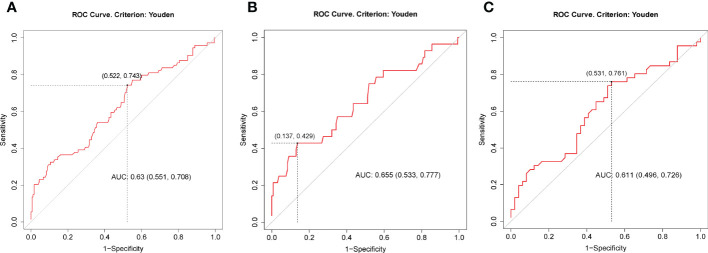
ROC curves for preoperative TSH as a predictor of gastrointestinal side effects of liraglutide among all patients **(A)**, males **(B)** and females **(C)**. The best cutoff points in **(A, B, C)** were 1.37 (specificity = 0.48, sensitivity = 0.74), 2.30 (specificity = 0.86, sensitivity = 0.43) and 1.33 (specificity = 0.47, sensitivity = 0.76), respectively. AUC, area under the ROC curve.

Binary logistic regression was performed to evaluate whether significant variables in univariate analyses were independent factors of GSEA. In the univariate regression model, variables including age (OR=1.02, 95%CI:1.00-1.05, p=0.037), male sex (OR=0.23, 95%CI:0.13-0.40, p<0.001), TSH (OR=1.75, 95%CI: 1.31-2.35, p<0.001), AGI (OR=2.45, 95%CI: 1.32-4.55, p=0.004), and concomitant gastrointestinal diseases (OR= 2.14, 95%CI: 1.10-4.19, p=0.026) were associated with GSEA (**Figure 1A**). In the final adjusted model, AGI use (adjusted OR=4.01, 95%CI: 1.90-8.45, p<0.001), gastrointestinal diseases (adjusted OR=3.29, 95%CI: 1.51-7.18, p=0.003), TSH (adjusted OR=1.79, 95%CI: 1.28-2.50, p=0.001) and male sex (adjusted OR=0.19, 95%CI: 0.10-0.37, p<0.001) were independently associated with GSEA occurrence (**Figure 1B**). Furthermore, ROC curve analysis showed that the continuous variable TSH was a predictor for GSEA in T2DM patients, with a 0.63 AUC and 1.37 cutoff point (**Figure 2A**). Grouping by sex, the AUC and cutoff point were both higher among male patients than female patients (0.66 vs. 0.61; 2.30 vs. 1.33, respectively) (**Figures 2B, C**). Furthermore, the mediating effects of TSH on independent variables (males, GID, AGI use, age) were assessed, and the results in **Table 2** showed that TSH had no mediating effect on males, GID, AGI use, age and GSEA (a=0.12, 0.16, 0.14 and 0.01, respectively. all p>0.05).

**Table 2:** Models of the mediating effects of TSH on the association between independent variables and GSEA.

	Independent variables	A	*p* value	B	*p* value	C’	*p* value
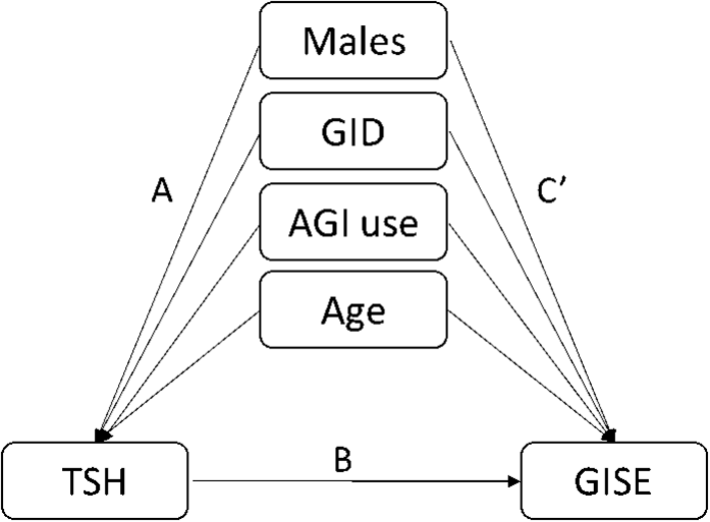	Males	0.12	0.083	0.16	<0.001	0.30	<0.001
	GID	0.16	0.305	0.15	<0.001	0.35	0.040
	AGI use	0.14	0.365	0.16	<0.001	0.34	0.002
	Age	0.01	0.170	0.15	<0.001	0.01	0.076

A is the indirect pathways from the corresponding independent variables to TSH, while path B links each TSH to GSEA. C’ is the direct pathways from the corresponding independent variables to GSEA. TSH, thyroid stimulating hormone; GID, gastrointestinal diseases; AGI, α-glucosidase inhibitor.

## 4 Discussion

Liraglutide is a human GLP-1 analog with 97% homology to native GLP-1 (3). Unlike endogenous GLP-1, which is rapidly degraded, liraglutide is a long-acting GLP-1 receptor agonist that is resistant to degradation. Large clinical trials designed to test the cardiovascular and renal safety of the new antidiabetic drug class of GLP-1 analogs surprisingly revealed that T2DM patients treated with liraglutide have fewer cardiovascular events than those treated with standard care (6) and do not affect renal function (12). By acting on GLP-1 receptors in the gastrointestinal tract and central nervous system, liraglutide delays gastric emptying, induces satiety, and reduces food intake, but it may also cause GSEA (20).

Here, we showed that the incidence of GSEA in patients treated with liraglutide was 29.13%, leading to the termination of patients with 4.33%, which was generally consistent with other studies (11). It is well known that the incidence of gastrointestinal effects in this study increased dose-dependently during the first 4 weeks of liraglutide treatment but declined thereafter (11, 14). Therefore, the observation period of this study was 4 weeks. An early study involving 4422 patients with T2DM revealed that non-metformin use and older age were associated with more significant GSEA leading to discontinuation of liraglutide treatment (17), but the results of this study did not find that metformin was different between the GSEA and non-GSEA groups. In univariate analysis, we also found that age was an influencing factor of GSEA. However, when adjusted for other variables, age became non-significantly associated with GISE outcomes. In addition, others reported that mild renal impairment may affect GSEA and cause them to be more frequently discontinued (21). In this study, SCr and AST were used as observation indicators of renal function and liver function, respectively. There was no significant difference in AST and SCr between the two groups, which may reflect the fact that the metabolism of liraglutide does not need to go through the liver and kidney. Thus, patients with mild to moderate liver and kidney impairment do not need to adjust the dose of liraglutide. In addition, although a previous study reported that the incidence of GSEA increased when combined with insulin treatment (22), HbA1c, insulin and C-peptide before administration did not affect the occurrence of GSEA in this study. It may also be worthwhile to investigate whether other glycemic profiles, including fasting glucose and insulin resistance, affect the GSEA to liraglutide.

Through logistic regression analysis, we found that the combination of AGI treatment, gastrointestinal diseases, TSH and female sex were independently tied to GISA. Similarly, Ren et al. (23) recently reported that liraglutide combined with acarbose increased the incidence of GSEA compared to liraglutide combined with metformin. The most common adverse reaction of AGI (acarbose) is also gastrointestinal side effects, with an incidence rate of approximately 17%, which is characterized by flatulence and abdominal distension (24). It is speculated that the combination of AGI and liraglutide may produce a superposition effect of gastrointestinal side effects. Therefore, it is necessary to choose other alternative hypoglycemic drugs or AGI use in small doses. Although gastrointestinal disease is not a contraindication to treatment with liraglutide, GLP-1 receptor agonists have the effect of delaying gastrointestinal emptying and may aggravate the disease (23). Therefore, it is suggested that patients with digestive tract disease should be treated with liraglutide with caution to avoid poor tolerance and compliance.

The safety of liraglutide in patients with thyroid diseases has been reported (25). Several studies have found that GLP-1 receptor agonists may cause thyroid C-cell adenomas and C-cell carcinomas in rodents (26, 27), and thus GLP-1 receptor agonists are contraindicated in those with a history of medullary thyroid cancer. However, the LEADER trial (28) in T2DM patients and a meta-analysis (29) excluded a major increase in the risk of total malignant neoplasms (including pancreatic and thyroid neoplasms) with liraglutide treatment. In the present study, we further evaluated the relationship between thyroid function and GSEA. Unexpectedly, high TSH is an independent risk factor for GSEA of liraglutide and might predict the occurrence of GSEA. Subgroup analysis showed that the TSH cutoff points for predicting GSEA were 1.33 and 2.30 in females and males, respectively. However, the mechanism by which TSH increases the occurrence of gastrointestinal events remains to be studied. Speculatively, activation of the TSH receptor in the gastrointestinal tract may affect gastrointestinal events. In addition, we also found that the incidence of GSEA in females (48.42%) was significantly higher than that in males (17.61%), and female sex was an independent risk factor for GSEA. Consistently, Long et al. (30) recently demonstrated that female sex and GLP-1 receptor single-nucleotide polymorphisms could be predictors of gastrointestinal adverse reactions with liraglutide in T2DM patients. Through mediation analysis, TSH had no mediating effect on sex and GISE, and possibly small sample size caused that. Some studies showed the apparent sex-specific differences in the frequency of GSEA partially attributed to higher exposures to liraglutide in females, possibly due to their lower average body weight (31) and the greater prevalence of functional GID among females (32). Therefore, it is necessary to take measures to prevent GSEA in women with type 2 diabetes treated with liraglutide.

The strengths of the study are that it is the first report on the association of serum TSH levels with the GSEA of liraglutide in patients with T2DM. Nevertheless, this study has several limitations. First, the symptoms of gastrointestinal side effects came from the patient’s subjective report at the time of treatment, which may introduce some bias. In addition, we did not evaluate the relationship between the variables and the interruption of liraglutide. It may also be worth exploring whether risk factors, especially TSH, would affect the therapeutic effect of liraglutide on glycemia and weight control. Third, this study had a relatively small sample size and incomplete follow-up. Multicenter and larger prospective studies are needed to confirm our findings. Final, the study included only Asians who are known to respond to incretin-based therapies differently from people of other ethnicities (33, 34).

In conclusion, the present study suggests that the combination of AGI, concomitant gastrointestinal diseases, sex and TSH level are independently associated with the GSEA of liraglutide in patients with T2DM. Caution should be taken in using liraglutide or other GLP-1 receptor agonist treatments treatment for patients with a combination of AGI, concomitant gastrointestinal diseases, high TSH levels and/or female patients due to being prone to GSEA.

## Data availability statement

The original contributions presented in the study are included in the article/supplementary material. Further inquiries can be directed to the corresponding authors.

## Ethics statement

The studies involving human participants were reviewed and approved by Ethics Committee and Institutional Review Board of the 910th hospital of PLA. Written informed consent for participation was not required for this study in accordance with the national legislation and the institutional requirements.

## Author contributions

WH: Conceptualization, Methodology, Software, Investigation, Formal Analysis, Writing - Original Draft. LZ: Methodology, Visualization, Investigation Review. CR: Data Curation, Writing - Original Draft. CQ and WM: Resources, Supervision. ZL and ZZ: Conceptualization, Funding Acquisition, Resources, Supervision, Writing - Review & Editing. All authors contributed to the article and approved the submitted version.
